# Role of Curing Conditions and Precursor on the Microstructure and Phase Chemistry of Alkali-Activated Fly Ash and Slag Pastes

**DOI:** 10.3390/ma14081918

**Published:** 2021-04-12

**Authors:** Marija Nedeljković, Bahman Ghiassi, Guang Ye

**Affiliations:** 1 Faculty of Civil Engineering & Geosciences—Materials & Environment, Delft University of Technology, Stevinweg 1, 2628 CN Delft, The Netherlands; G.Ye@tudelft.nl; 2 Centre for Structural Engineering Design and Informatics, Faculty of Engineering, University of Nottingham, Nottingham NG7 2RD, UK; bahman.ghiassi@nottingham.ac.uk

**Keywords:** alkali-activated materials, reaction products, chemical composition, curing conditions, PARC

## Abstract

Understanding the role of curing conditions on the microstructure and phase chemistry of alkali-activated materials (AAMs) is essential for the evaluation of the long-term performance as well as the optimization of the processing methods for achieving more durable AAMs-based concretes. However, this information cannot be obtained with the common material characterization techniques as they often deliver limited information on the chemical domains and proportions of reaction products. This paper presents the use of PhAse Recognition and Characterization (PARC) software to overcome this obstacle for the first time. A single precursor (ground granulated blast-furnace slag (GBFS)) and a binary precursor (50% GBFS–50% fly ash) alkali-activated paste are investigated. The pastes are prepared and then cured in sealed and unsealed conditions for up to one year. The development of the microstructure and phase chemistry are investigated with PARC, and the obtained results are compared with independent bulk analytical techniques X-ray Powder Fluorescence and X-ray Powder Diffraction. PARC allowed the determination of the type of reaction products and GBFS and FA’s spatial distribution and degree of reaction at different curing ages and conditions. The results showed that the pastes react at different rates with the dominant reaction products of Mg-rich gel around GBFS particles, i.e., Ca-Mg-Na-Al-Si, and with Ca-Na-Al-Si gel, in the bulk paste. The microstructure evolution was significantly affected in the unsealed curing conditions due to the Na^+^ loss. The effect of the curing conditions was more pronounced in the binary system.

## 1. Introduction

Alkali-activated materials (AAMs) are multi-phase materials composed of crystalline and amorphous phases, which together with alkalis and water react over time [1]. This is followed by a net decrease in volume due to chemical shrinkage, i.e., the volume of the reaction products is less than the initial volume of the unreacted particles and the liquid. The principal reaction product in alkali-activated ground granulated blast-furnace slag (GBFS) is calcium (-sodium) aluminosilicate hydrate gel (C-(N-)A-S-H) [2,3], while it is sodium aluminosilicate hydrate gel (N-A-S-H) in alkali-activated fly ash (FA) or metakaolin [4]. 

The microstructure of AAMs, i.e., the reaction products and unreacted particles, their chemical composition, amounts, and distribution, makes the fundamental link between the hardening and long-term properties [5]. The changes in microstructure features over time and the chemical composition of the reaction products in AAMs have been the focus of many previous studies [6,7,8,9,10]. Despite numerous efforts to characterize AAMs in the literature, rigorous definitions on the reaction products (N-A-S-H, C-A-S-H, C-(N-)A-S-H) and possible secondary products, such as zeolites and layered double hydroxides, in these materials do not exist yet [6,10,11]. This is due to the complexity of the chemistry (element distribution gradients within phases) and microstructural features of AAMs (phase distribution). 

The presence of alkalis in the system makes defining the reaction products even more complicated. This is because alkalis can exist in several forms in Ca-rich AAMs (i.e., incorporated into C-S-H, physically adsorbed on the surface of reaction products, and free in the pore solution) [12]. The presence of Al in the C-A-S-H gels enhances the uptake of alkalis in the system compared to pure C-S-H gels. Each Al in the gel requires an alkali for achieving the charge balance with respect to Si^4+^. The extent to which the charge balance is achieved, therefore, depends on the available alkalis. However, the binding capacity of alkalis itself depends on the amount of Si replaced by Al. In AAMs, due to the higher Al content available from precursors (FA, GBFS) and due to the use of highly alkaline activators, the uptake of alkalis in the gels is expected to be high. The identification of different gels may be complex when binary mixtures are used, e.g., FA and GBFS. The spatial distribution of different gels can be correlated with the dissolution of FA and GBFS particles, which in turn gives insight into the reaction mechanism of the pastes. Therefore, experimental characterization of the spatial distribution of different gels and associated chemical compositions is important. Additionally, understanding the long-term performance of AAMs requires knowledge on the relative contribution of FA and GBFS to the gel formation, which is not obtained easily with the current methods.

The main bulk of experimental characterization techniques in materials science, such as X-ray diffraction (XRD), Fourier-transform infrared spectroscopy (FT-IR), Thermogravimetry (TG), Nuclear magnetic resonance spectroscopy (NMR), Small-angle neutron scattering (SANS), and Field Emission Scanning Electron Microscopy (FE-SEM)-Energy Dispersive X-ray (EDX), have been used extensively to characterize the chemical and spatial characteristics of AAMs [3,13,14,15]. SEM-EDX can provide both local and bulk analyses, which are both important for studying multiphase materials. In general, bulk characterization with Spectral Imaging (SI) of the samples with SEM-EDX has not been utilized to its full potential because of the lack of techniques and algorithms for deriving phase compositions and distributions from SI data sets. 

This is partly because element overlay maps from SI are limited to combinations of a maximum of three elements if unique colour coding per phase is required for visualization of phase distributions. Distinguishing phases based on three elements only is too restricted for analysis of multi-element materials like AAMs. With other algorithms, such as Principal Component Analysis, the principal components and phases do not necessarily coincide. For a more extensive discussion on this topic the reader is referred to Van Hoek et al. [16]. 

A possibility for overcoming the shortcomings of commercial SI-based phase recognition tools in the characterization of multiphase materials is the PhAse Recognition and Characterization (PARC) software developed by Van Hoek et al. [16], which is a postprocessing software for SI data. This software has been previously used for the characterization of cultural heritage objects [17], dust characterization [18], and the microstructure of metallurgical slag [16,19]. 

In this paper, we use it, for the first time, for the microstructural and chemical characterization of AAMs cured in different conditions and for different times.

The technique is used to distinguish the unreacted particles (FA and GBFS) and the reaction products in two alkali-activated pastes. The mixtures included a single precursor system (alkali-activated GBFS, referred to as S100) and a binary precursor system (alkali-activated GBFS + FA, with 50%:50% weight ratio, referred to as S50). The degree of reaction (reacted fraction) of FA and GBFS in the pastes are also determined and presented. The results obtained from PARC are compared and confirmed with the independent bulk analytical techniques (X-ray Powder Fluorescence and X-ray Powder Diffraction) ).

## 2. Materials and Methods

### 2.1. Materials and Sample Preparation

The raw materials used in this study were the GBFS from ORCEM, Moerdijk, and the FA from VLIEGASUNIE BV, Culemborg, the Netherlands. The GBFS had a specific gravity of 2.9 g/cm^3^, while the FA had a specific gravity of 2.44 g/cm^3^. The chemical composition of the GBFS and FA was determined with X-ray Powder Fluorescence (XRF) and is given in Table 1. The XRF measurements were done with Panalytical AXIOS Max Advanced XRF spectrometer. The XRF analysis of raw materials were performed with fused beads and lithium tetraborate/methaborate as a flux. XRF bead analysis is not suitable for sulphur analysis. Therefore, sulphur (S) was determined with the Eltra Sulphur analyser. The loss on ignition (LOI) was determined with the LECO Thermographic Analyser (TGA701). The negative LOI for the GBFS (Table 1) was related to the oxidation of the sulphur-rich species in the GBFS. It should be noted that the LOI was not corrected in the XRF measurements. 

**Figure 1:**
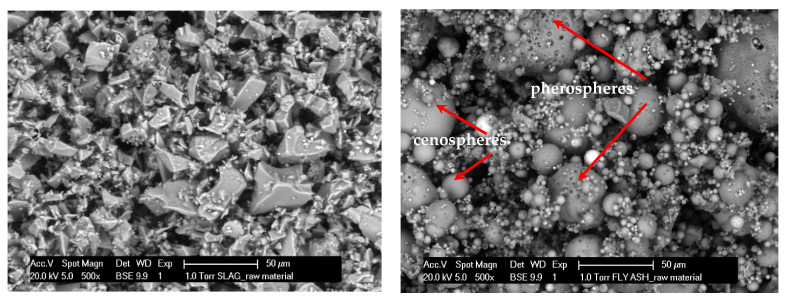
ESEM-BSE images of raw ground granulated blast-furnace slag ((GBFS) (**left**)) and fly ash ((FA) (**right**)) particles.

The particle shape of GBFS and FA was studied with SEM in backscattered electron mode. The raw GBFS particles have clear edges and angles, as shown in Figure 1. On the other hand, raw FA particles consist of individual and agglomerated glassy spheres of different sizes. A large quantity of FA spheres is hollow, known as cenospheres or floaters, which are very light and tend to float on water surfaces [20]. FA also contains small spherical particles within a large glassy sphere, called pherospheres [20], as indicated by the red arrows in Figure 1. The external surfaces of the solid and hollow spherical particles of low-CaO FA, as the FA in this study, are generally smoother than those of high-CaO FA, which may have surface coatings rich in CaO [20]. 

**Figure 2:**
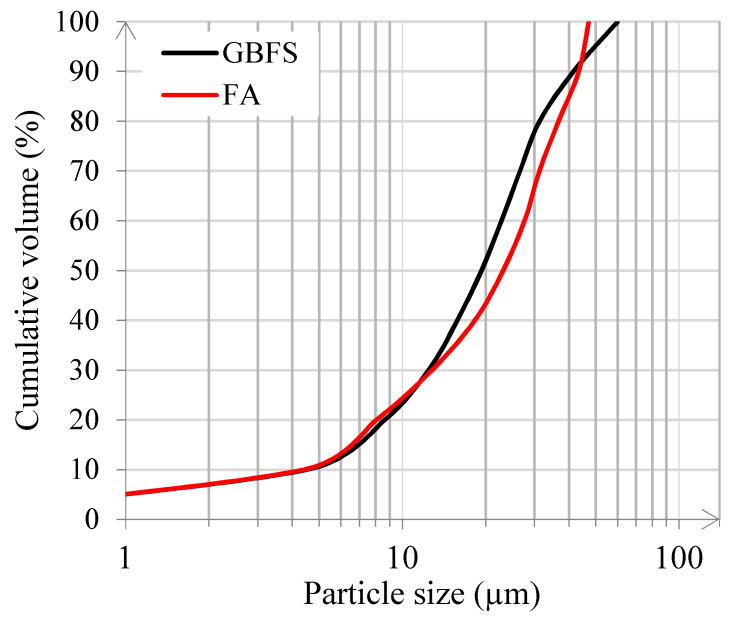
Particle size distributions of the studied GBFS and FA measured with Laser diffraction analyser.

Figure 2 shows the particle size distributions of the GBFS and FA, which were measured with the EyeTech Laser diffraction analyser. An external ultrasonic bath was used for the deagglomeration of the particles in order to increase the dispersion efficiency. The d_50_, which represents the particle size of the group of particles, was 19 μm for the GBFS, while for the FA, d_50_ was 21 μm. According to the literature, the dissolution of the GBFS is dominated by small particles. Particles with a size >20 μm react slowly, while particles with a size <2 μm react completely after 24 h in blended cement and alkali-activated binders [21,22].

**Figure 3:**
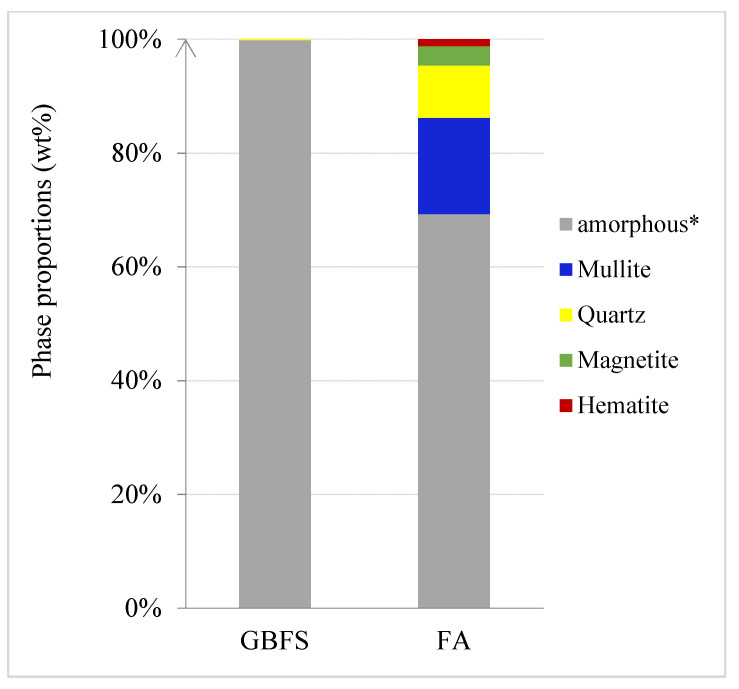
Quantitative phase analysis with the Rietveld method for the unreacted GBFS and FA. * stands for proportion of amorphous phase when excluding the internal standard in QXRD calculations.

X-ray diffraction (XRD) is used to study the minerology of raw materials. Figure 3 shows the quantitative phase analysis with the Rietveld method for the GBFS and FA. It can be seen that the GBFS is fully amorphous, while the FA is a mixture of amorphous (69 wt%) and crystalline phases, such as quartz, mullite, magnetite and hematite. 

Before the preparation of the pastes, the alkaline activator was prepared by mixing anhydrous pellets of sodium hydroxide with deionized water and commercial sodium silicate solution (27.5 wt% SiO_2_, 8.25 wt% Na_2_O). After mixing, the activator was kept in the laboratory conditions with a temperature around 20 °C to cool down for 24 h prior to the paste mixing. The activator Na_2_O concentration was 4.8 wt% with respect to the precursor mass (FA + GBFS). For each paste, the liquid to binder mass ratio was 0.5. The pastes were produced with the following FA/GBFS ratios of 50:50, 0:100 wt%, named S50, S100, respectively (Table 2).

The mixing time for producing alkali-activated pastes was 5 min. The precursors (FA and GBFS) were dry-mixed for 3 min and then mixed with the activator. The mixing continued for the next 2 min until the moment when the mix was homogenized. The pastes were cast in cylindric polyethylene jars with a 35 mm diameter and a 70 mm height and vibrated for 15–30 s on a vibrating table. The samples were stored in the closed jars for 24 h after casting. For unsealed cured conditions, samples were removed from the jars and cured in a curing room at room temperature and relative humidity (RH) of ~99% for 28 days afterwards. The sealed samples were kept in the jars in the same curing room as the unsealed samples until the testing. The sealed samples were cured for up to 1 year. The samples were characterized with PARC, XRF, and XRD after certain periods with pre-drying (1, 7, 28 days, 1 year). For PARC, XRF, XRD, the representative samples, after a certain period of curing, were gently crushed (from the sample surface) into small pieces with dimensions of 1–2 cm^3^ and then immersed in isopropanol for one week, by which water is first replaced and then evaporated. Subsequently, the samples were placed under vacuum at 25 °C for at least three weeks prior to testing. After predrying, the samples, for SEM-EDX analysis, were impregnated using a low-viscosity epoxy resin and then polished down to ¼ μm. Before performing SEM-EDX, a carbon coating was applied to the polished sections of the samples.

### 2.2. Characterization Methods

#### 2.2.1. SEM-EDX, PARC, IGOR

The characterization methodology is based on the following steps:

- Spectral Imaging (SI) of the samples with SEM-EDX,
- evaluating the SI images with PARC software to define phases, their area/volume percentages, and spatial distribution,
- calculation of bulk chemical compositions of the phases and consistency check with the bulk composition from XRF analysis.

**Scheme 1:**
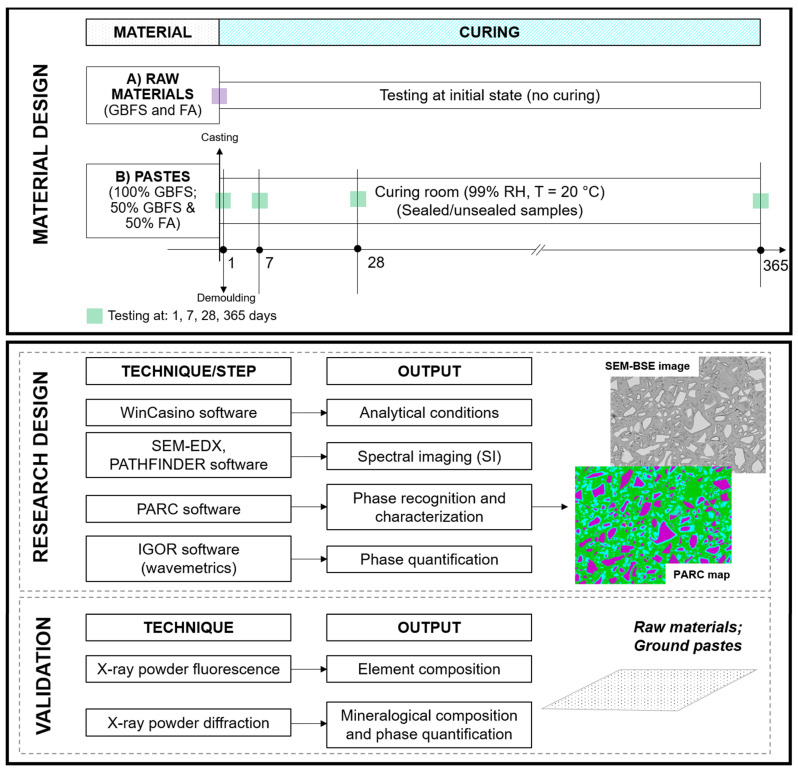
Overall research flow.

A general description of each of these steps is given next with illustration in Scheme 1.

##### SEM-EDX

To study the paste microstructure, a JEOL JSM-7001F FE-SEM equipped with two EDX detectors (30 mm^2^) and the Thermo Fisher Scientific NORAN System 7 (NSS.3.3) microanalysis software was used. 

**Figure 4:**
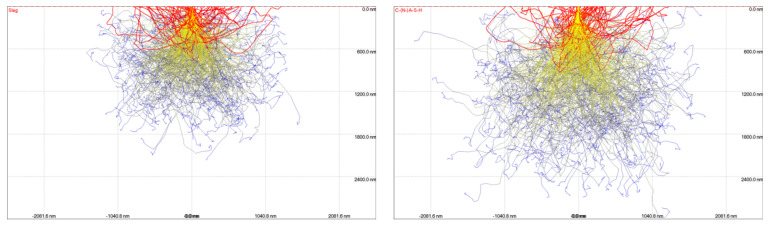
Monte Carlo simulation of the penetration of 1000 electrons accelerated at 15 kV in a beam radius of 10 nm into a GBFS (**left**) and the C-(N-)A-S-H gel (**right**). The red trajectories are back-scattered electrons, which result from elastic scattering events. Inelastic scattering events cause a reduction of electrons’ energy until the eventual disappearance in the specimen bulk. Yellow trajectories represent high-energy trajectories, and blue represents low-energy trajectories.

The optimal microscope conditions for microanalysis were determined with Monte Carlo simulation in WinCasino v2.41 software (www.gel.usherbrooke.ca/casino/index.html accessed on 11 April 2021), using the experimental density (2.9 g/cm^3^ for GBFS and 2.6 g/cm^3^ C-(N-)A-S-H gel) as an input parameter [11]. Based on several iterative experiments, 15 kV was chosen as the optimum beam accelerating voltage. At a beam radius of 10 nm (*X*-axis), Figure 4 shows the maximum penetration depth (*Y*-axis) of the electron trajectories as determined by Monte Carlo simulation, ranging from 0.6 to 1.8 μm for the GBFS particles and from 1.0 to 2.5 μm for the C-(N-)A-S-H gel. For backscattered electrons, the maximum sampling depth was about 30% of the interaction volume depth, and its lateral dimension was close to the interaction volume depth. As electrons penetrate deeper, the lateral spread of the electron-solid interaction region increases. The lateral dimension of the interaction volume for cement-based materials is thought to be around 1–2 μm [23], which can be taken as the chemical spatial resolution for the SI.

**Figure 5:**
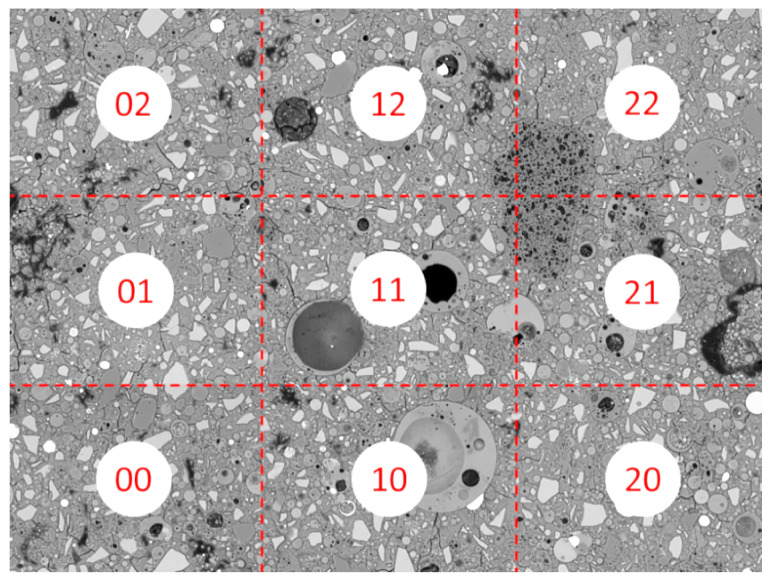
SEM-BSE image of the area of interest (3 × 3 matrix (fields are labelled 00–22), with an individual SI field comprising of 512 × 384 pixels) for paste microstructure characterization (sample is paste S50, unsealed cured for 28 days). All 9 fields of the area were analysed under the same analytical conditions. The total field width equals 750 microns.

The microscope and EDX settings were kept constant for all the samples. For the microscope, the following settings were used: accelerating voltage 15 kV, beam current 3.4 nA, magnification 500× (equivalent to a field width of 256 microns), and a working distance (is the distance between the pole piece and the specimen surface) of 11.5 mm. The video images were collected with a resolution of 1024 × 768 (pixel size equal 0.25 μm). For EDX data collection, the resolution of each SI dataset was 512 × 384 (pixel size equal 0.5 μm) with a total acquisition time of 3600 s. An example of a stitched image of nine fields in SEM-backscattering electron (BSE) mode is presented in Figure 5. The 3 × 3 matrix (00-22) is selected so that the phase distributions over a larger sample area can be determined. Finally, SEM-EDX analysis provides data on the chemical composition across the large field of the sample by spectral imaging. 

##### PhAse Recognition and Characterization (PARC) Software

The PARC software was used to evaluate the SI data files. After loading the SI data file into the PARC software, from each individual pixel spectrum, a user-defined number of channels covering the energy range of interest is used for evaluation of the peak position and height in each spectrum. The collected spectra are classified into empty, embedding, pure phase and mixed spectra (e.g., from a phase boundary) based on the detected peaks exceeding a user-defined threshold value. Empty spectra contain no peaks, embedding spectra only peaks from epoxy or conductive resin, and all other spectra contain meaningful spectral information. The next step is grouping the spectra with identical peak combinations and designating these as PARC phases [16].

Using this procedure, all individual pixels are assigned to different phase groups. Once a particular setup (phase model) is defined, this model can be applied to multiple SI-datasets collected using the same analytical conditions.

**Figure 6:**
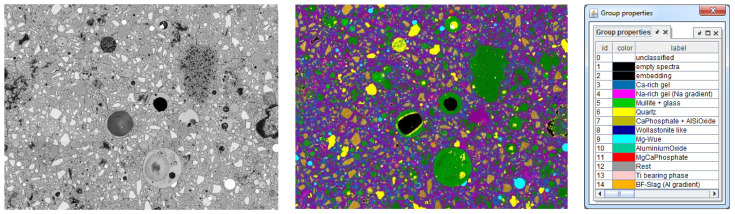
BSE image of the sample (**left**) and its corresponding PhAse Recognition and Characterization (PARC) phase map (**middle**). PARC legend (**right**) of different phases (groups) defined for the paste S50 (unsealed cured for 28 days) for the phase map (middle image).

Figure 6 presents an example of the PARC phase map of a SI dataset of the microstructure (sample is paste S50, unsealed cured for 28 days). The phase model obtained for the first field (00 field, see Figure 5) can be applied to other fields, provided these were acquired under the same analytical conditions. If all the pixels in the new field are recognized with the first phase model, the next field can be processed. In case the second field contains pixels that are not recognized using the phase model, they will be assigned to new PARC phases, and the PARC model will be updated accordingly. This continues until all pixels in all 9 fields are satisfactorily assigned (see Figure 5). When the final phase model is defined, the PARC phase area proportions and the phase spectra results are exported. Spectra are processed using NSS microanalysis EDX software. Standardless EDX analysis was performed. The Phi-Rho-Z matrix correction procedure was used for raw data processing without normalizing the EDX results to 100%. The quantification in the Phi-Rho-Z mode is an element quantification method based on the matrix correction with the depth distribution function (Phi), mass density (Rho), and mean atomic number (Z). An example of a PARC legend of different phases (groups) is shown in Figure 6 (right) for the paste S50.

##### IGOR

In addition to PARC, IGOR PRO 7 was used for the quantification of the mass percentage of each phase in the samples. IGOR is a statistical program with mathematical and image processing functions (www.wavemetrics.com (accessed on 11 April 2021)) that is used in combination with PARC to:

- combine the information obtained from multiple fields and obtain the variance between fields (standard deviations in area proportions of phases),
- calculate average phase chemistry for one or multiple fields,
- calculate average/sum area % per phase for multiple fields,
- calculate sample bulk chemistry (mass balance) using densities of the unreacted FA and GBFS (known) and reaction products (gel).

To convert the phase areas (obtained from PARC) to phase mass percentages, the density of each phase (gel and unreacted FA and GBFS) is required. As presented before, the FA and GBFS densities used in this study are 2.44 g/cm^3^ and 2.97 g/cm^3^, respectively. The densities of gels were adopted from the study of Thomas et al. [24]. The densities were determined using a neutron-scattering method in conjunction with a hydration model [24]. For both pastes S50 and S100, three main gels were identified. Since it is very difficult to determine gel densities experimentally, the gels’ densities from the literature were then assigned to the gel phases in order to convert the PARC gel area measurements to phase proportions (wt%). 

##### Degree of Reaction

The degree of reaction of GBFS and FA is calculated by comparing the volume fraction (V_t_) of unreacted materials (GBFS in paste S100 and GBFS + FA in paste S50) with the volume fractions prior to the mixing of raw materials with an alkaline activator (at time zero, V_0_). 

Based on the stereology principles [25], the area fraction of unreacted GBFS and FA in a 2D image is equal to the 3D volume fraction. The area fractions of unreacted GBFS and FA were obtained from multiple fields (the representative fields for each curing time are shown in Figures 15a–f-ii and 20a–f-ii) in the PARC analysis. The degree of reaction of GBFS or GBFS and FA (α (t)) is then calculated as:

(1) $$\alpha \;\left(t\right)=\left(1-\frac{V_{t}}{V_{0}}\right)\cdot 100\%\;$$

Volume fractions of the GBFS and the alkaline activator at the time zero (V_0_) in paste S100 were determined from the initial liquid-to-binder ratio. The volume of the activator mixed with 1 kg of GBFS was calculated as 0.4 L (activator density was measured as 1.25 g/cm^3^, and 0.5 kg of activator was added). Using the GBFS density (2.97 g/cm^3^), 1 kg GBFS corresponds to 0.337 L. Hence, the volume fraction of GBFS at the time zero (V_0_) for paste S100 was 45.73%. For the paste S50, the same calculations were made, considering the density of FA (2.44 g/cm^3^) and GBFS. The volume fractions of GBFS and FA at the time zero (V_0_) for paste S50 were 22% for GBFS and 26% for FA.

#### 2.2.2. X-ray Powder Fluorescence

The paste samples were ground to powder and subsequently pressed under high pressure (20 tonnes) into a tablet to obtain a homogeneous sample surface for measurements. When making tablets a dilution using wax was performed with a dilution factor 1:4. The pressed tablets were analyzed with X-ray fluorescence spectrometer (Axios Max WD-XRF, Malvern Panalytical Ltd). Analysis of the XRF data was performed with SuperQ5.0i/Omnian software.

#### 2.2.3. X-ray Powder Diffraction

XRD measurements were performed on powered samples. A few grams (3–5 g) of samples were ground to below a fineness of 15 µm, with an internal standard of 10 wt% added metallic silicon. Both the sample and the internal standard were premixed and ground for 20 min under cycloxhexane (~7 mL) using sintered corundum grinding elements with a McCrone micronizer mill. Afterwards, the slurry was poured into a ceramic dish and transferred to an oven. The slurry was kept for a few minutes at 65 °C in the oven. Subsequently, the dried powder was pressed in a bottom-loaded XRD holder and prepared for XRD measurement. XRD diffractograms were acquired from 10° to 130° 2-theta with a Bruker D4 diffractometer using Co-Kα radiation and a Lynxeye position-sensitive detector. The Bruker Topas software was used to perform the Rietveld quantification of the phases. The Rietveld fitting error obtained on the amorphous phase in the samples showed a high-precision with general statistical errors of less than 1.0% absolute.

## 3. Results and Discussion

### 3.1. Characterization of Raw Materials with PARC

#### 3.1.1. Raw GBFS

**Figure 7:**
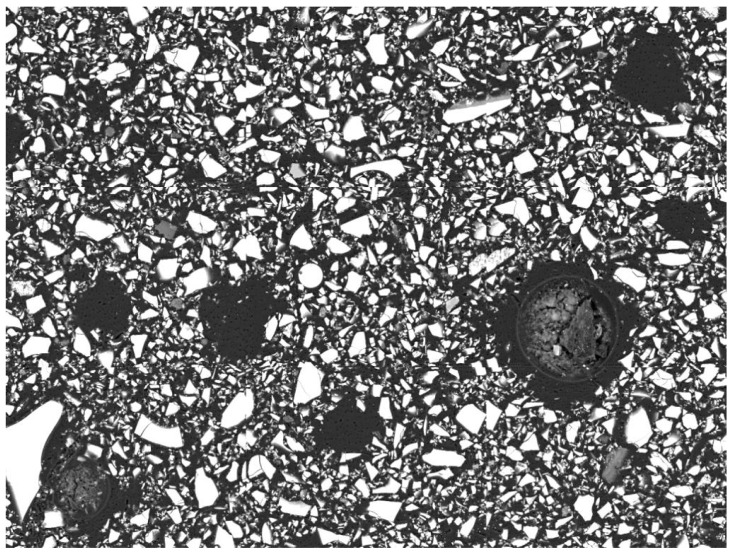
Large area BSE image of a polished section of an epoxy-impregnated unreacted GBFS (the number of acquired fields was 9). The image width is 768 µm.

The representative BSE image of raw GBFS was divided into nine fields, as shown in Figure 7. Subsequently, a data set from each SI image field was selected and imported into PARC for the preliminary phase characterization according to the procedure described in Section 2.2.1. 

**Figure 8:**
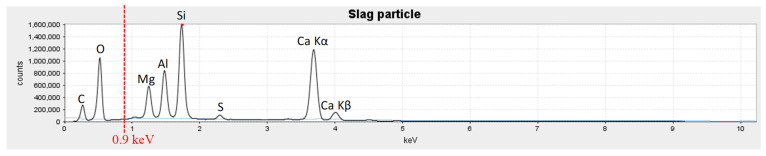
PARC sum spectrum for a GBFS particle (purple coloured in the PARC map, Figure 9), indicating the presence of Mg, Al, Si, S, and Ca by their characteristic X-ray lines.

The phases were distinguished by using up to seven X-ray peaks (each peak corresponding to an element, exception was Ca, where Kα and Kβ can both be above threshold) exceeding the selected threshold value, as shown in Figure 8. Here, the user-defined threshold (minimum energy cutoff) was selected as 0.9 keV (so, C and O are not used for phase definition). The sum EDX spectrum of GBFS pixels is presented in Figure 8. 

**Figure 9:**
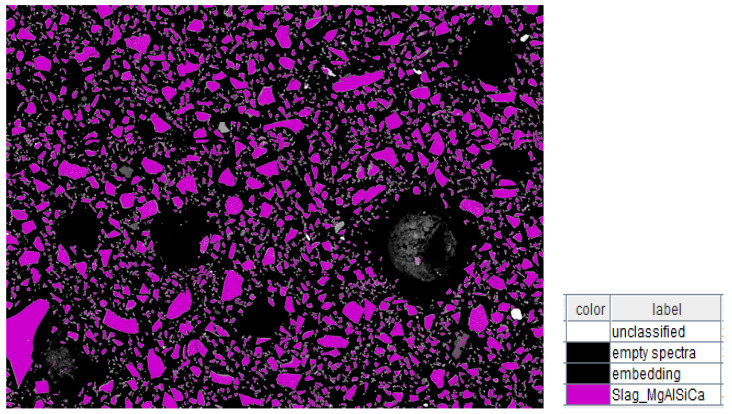
Large-area PARC phase map of the unreacted GBFS ((**left**) (image width: 768 µm)) and the legend of group phases identified in the unreacted GBFS (**right**).

Figure 9 shows the large-area PARC phase map of the raw GBFS and its corresponding phase legend, where the GBFS particles are coded as purple.

The composition of GBFS particles, which was obtained by averaging the field compositions with IGOR, is presented in Table 3. A comparison of the GBFS bulk composition obtained from XRF (see Table 1) and PARC (see Table 3) shows close correspondence or deviations that can be explained by Fe-metal droplets (yellow circled in Figure 9) and Al_2_O_3_ grains (white circled in Figure 9) included in the slag samples measured with XRF. These are naturally occurring contaminants in GBFS.

#### 3.1.2. Raw FA

**Figure 10:**
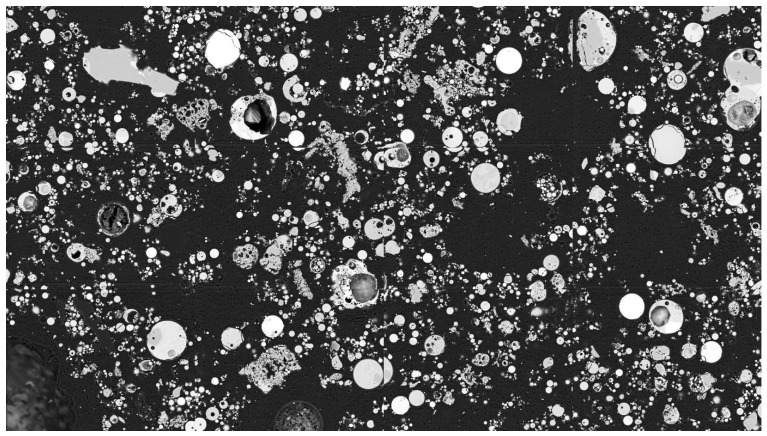
Large-area BSE image of a polished section of an epoxy-impregnated unreacted FA (the number of acquired fields was 12). The image width is 1024 µm.

Since FA is a more heterogenous material compared to GBFS, its representative area was divided into 12 fields (Figure 10). 

**Figure 11:**
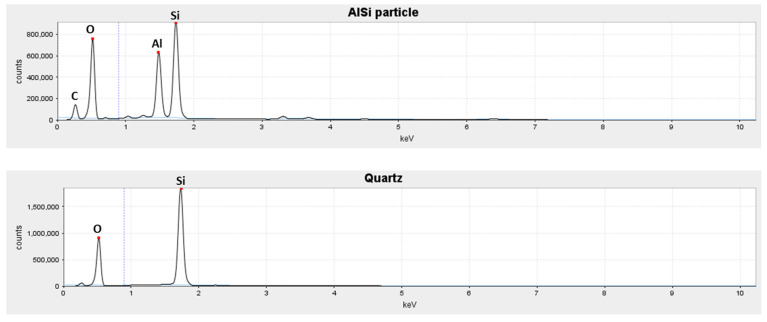
PARC sum spectra for AlSi pixels (blue coloured in the composite map, Figure 12) and Quartz (SiO_2_, yellow coloured in the composite map, Figure 12), indicating the presence of O, Al, and Si by their characteristic X-ray lines.

**Figure 12:**
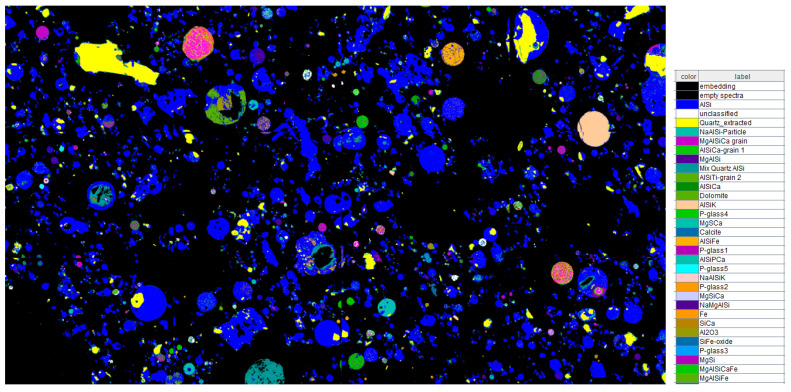
Large-area PARC phase image with individual phases in unreacted FA (width 1024 µm) (**left**); legend of identified phases (**right**).

The EDX spectra for the AlSi and quartz phases, as derived from PARC, are presented in Figure 11 since these are the major phases in the FA mineralogical composition. Again, the procedure followed for the phase map’s construction was as explained in Section 2.2.1. This procedure generated the phase map presented in Figure 12 for the BSE image shown in Figure 10. 

The distribution of the identified phases in FA particles (Figure 12) shows that the main phases are mullite and AlSi glass (blue colour, denoted as AlSi in the legend), which significantly varied in surface area, and quartz (yellow colour), with the corresponding spectra shown in Figure 11. Hematite (red colour), Na, P, Ti, Ca, Mg-alumina silicates, and some impurities, such as calcite and dolomite, were also identified. The corresponding phase compositions and their proportions are presented in Table 4. The bulk composition of FA obtained with PARC is presented in Table 5. It can be seen that PARC data (Table 5) agrees well with XRF data with regard to the FA bulk composition (Table 1). Furthermore, PARC reproduces the quartz and hematite fractions (see Table 4) obtained with the Rietveld method very well (see Figure 3).

Additionally, Figure 10 and Figure 12 show how phases can be enclosed differently. For instance, the quartz phase (yellow, Figure 12) is embedded in the AlSi phase (blue, Figure 12). A similar distinction was also made by Rickard et al. [26], who observed that quartz could be present as a discreet particle (larger crystallite size (>100 nm)) or within FA spheres (smaller crystallite size (<100 nm)). This means that a single particle can contain phases with different glass compositions and, therefore, different reactivities of each glassy phase within particle can be expected, as shown in the literature [27,28,29]. 

### 3.2. Characterization of Pastes with PARC

#### 3.2.1. GBFS Paste (S100)

The first step is to distinguish the unreacted GBFS particles and the reaction products. However, this is not an easy task due to the similarity of the chemical compositions of these phases. Therefore, it is necessary to subdivide the groups with identical combinations of elements into different subgroups corresponding to different phases based on the elemental proportions. This can be done in PARC by interactively selecting data regions in bivariate histogram plots or density plots of channel intensities [16] and looking for distinct populations. 

**Figure 13:**
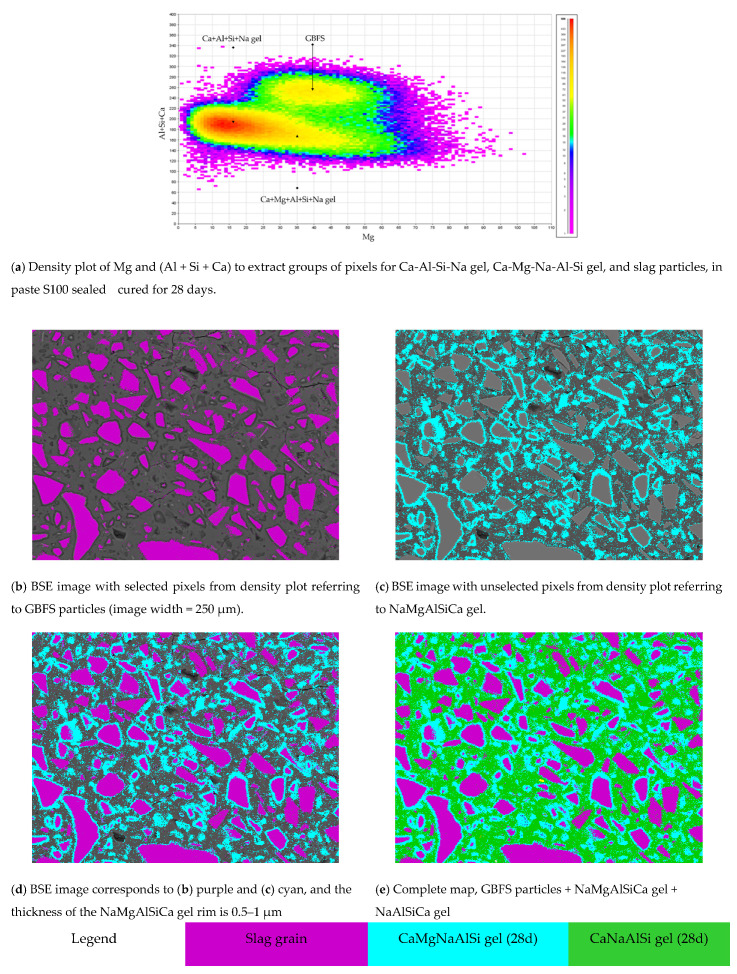
SEM-BSE images of the S100 microstructure (sealed cured paste for 28 days) and the compound domains of different phases.

The compositional variation in Mg and Al + Si + Ca content is used for distinguishing the phases. As an example, Figure 13a shows regions identified for unreacted GBFS particles and the reaction products of Ca-Al-Si-Na gel and Ca-Mg-Al-Si-Na gel of the sealed 28 days cured S100 paste in a so-called PARC density plot. These regions are used for mapping the phases in the BSE images (Figure 13b–e). The arbitrary boundaries in the density plot of Figure 13a were set to determine the corresponding volume fractions of Mg-rich and Mg-poor gels. Although these boundaries are arbitrarily chosen, they allow the quantification of corresponding volumes of gels. 

**Figure 14:**
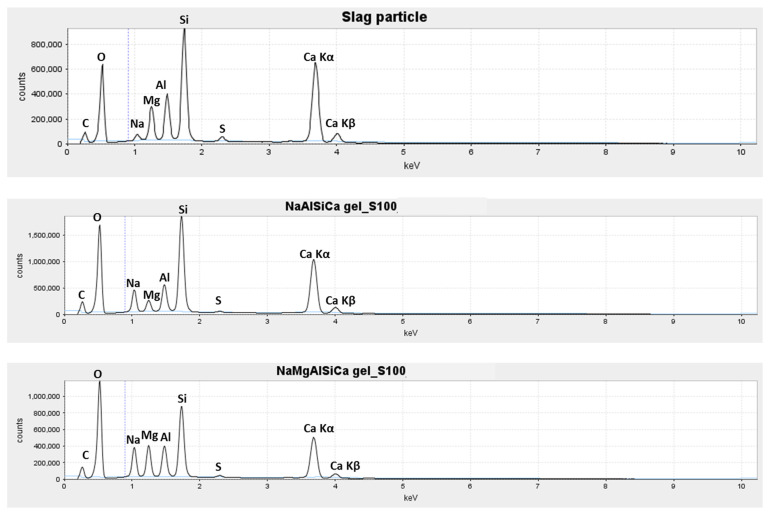
PARC sum spectra for compound domains of slag particles, NaAlSiCa gel, and NaMgAlSiCa gel in paste S100.

As it can be seen from the density plot in Figure 13a (and also from the spectra in Figure 14), there is a continuum in gel compositions from Mg-poor to Mg-rich, as well as a continuum of GBFS’s composition from Mg-poor to Mg-rich. In Figure 13d, the identified GBFS particle ((b) purple phase) and Ca-Mg-Al-Si-Na gel ((c) cyan phase) are presented. The complete map of phases in specimens sealed cured for 28 days (with the three main phases of Slag particles, Na-Al-Si-Ca gel, and Na-Mg-Al-Si-Ca gel with chemical compositions in Figure 14) is shown in Figure 13e. 

**Figure 15:**
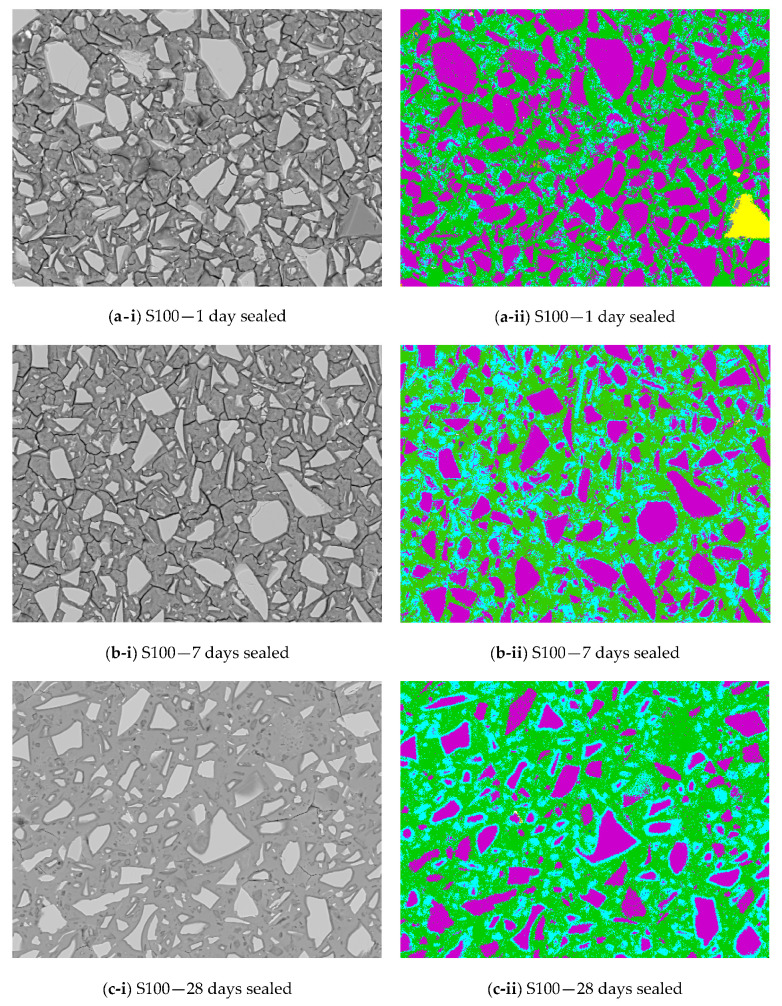

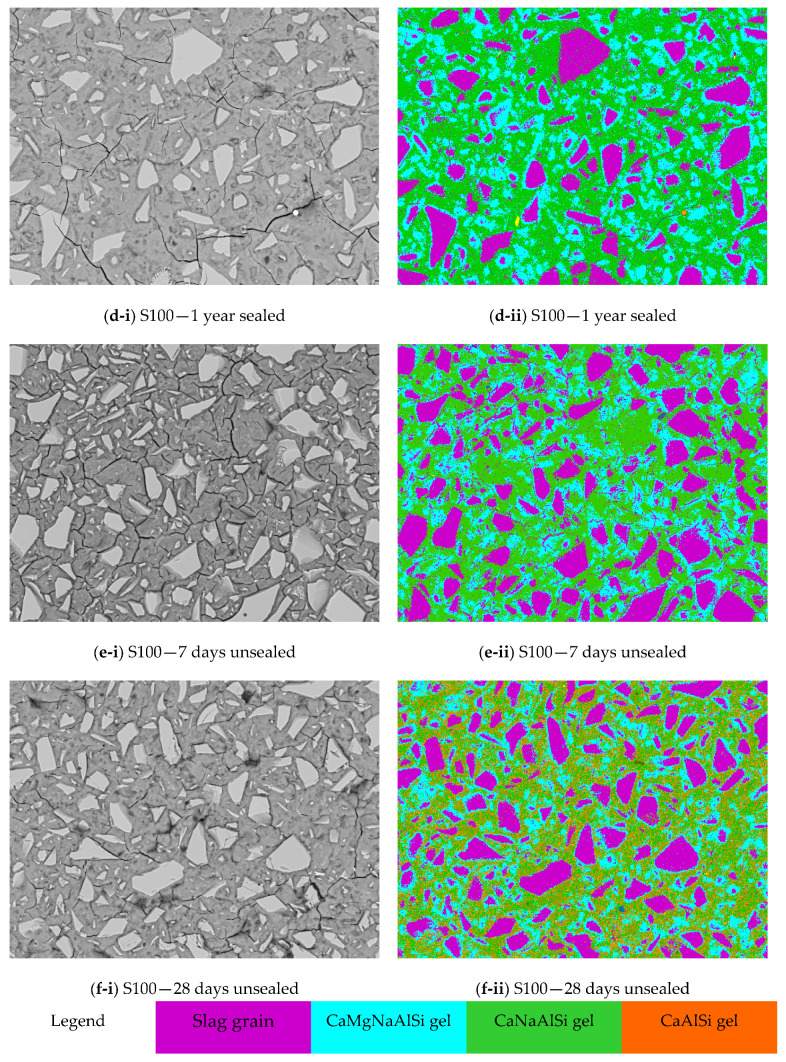
SEM-BSE images (**a–f-i**) of of paste S100’s microstructure at different curing periods, with mapped compound domains of the different phases (**a–f-ii**). Image width = 250 µm.

The phase distributions of the paste S100 as a function of curing time and curing conditions are visualized in Figure 15. Backscattered electron images of polished sections are shown in Figure 15a–f-i. The corresponding PARC maps are presented in Figure 15a–f-ii. The atomic ratios for the three identified phases (CaNaAlSi, CaMgNaAlSi, and CaAlSi) are presented in Table 6.

It can be observed that the major changes in time are in the Na/Si, Mg/Si, and Ca/Si element ratios. The Ca/Si ratio in CaNaAlSi gel of sealed S100 pastes is 0.61 at 1 day. This value increases to 0.84 from 7 to 28 days. Similarly, the Ca/Si atomic ratios were also observed in the hydration products of alkali-activated slag by Ben Haha et al. [30]. After 28 days, the Ca/Si ratio of NaAlSiCa gel did not change significantly (at 1 year Ca/Si is 0.85, Table 6). The fact that Ca/Si does not change after 28 days is evidence of limited GBFS dissolution in studied conditions.

Simultaneously, a transitional phase develops (CaMgNaAlSi gel) in the boundary of GBFS particles, which also did not change significantly between 28 days and 1 year. Meanwhile, the Ca/Si ratio of CaNaAlSi gel in unsealed S100 pastes was 0.61 at 1 day and increased to 0.69 (a lower value than that of sealed cured samples) from 7 to 28 days.

**Figure 16:**
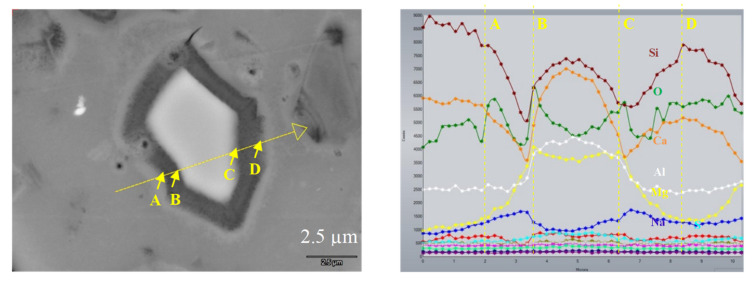
The BSE image of the microstructure around a reacted GBFS particle of the paste S100 after 1 year of reaction (**left**) and corresponding EDX linescan-profiles along the yellow arrow for the constituent elements (Si, O, Ca, Al, Mg, Na) (**right**).

The Al/Si ratio is significantly higher in CaMgNaAlSi gel (0.36–0.52) than in CaNaAlSi gel (0.25–0.27). The reason is that Al, similar to Mg, stays in the rims of the GBFS particles (see Figure 16) rather than in the outer gel. The measured Al/Si ratios (0.25–0.27) of CaNaAlSi gel in sealed cured samples were consistent with literature [31,32,33]. The Al/Si ratio increases over time in sealed conditions until 28 days, after which no changes are observed until 1 year. The Al/Si ratio values of unsealed cured samples were comparable to that of sealed cured samples.

The BSE images can also be used for investigating the evolution of gel chemistry around the reacted GBFS particles. As an example, the BSE image of a GBFS particle (S100 paste cured for 1 year) and the corresponding EDX linescan-profile at section A–D are shown in Figure 16. In the GBFS particle boundary, a transition zone is established for a CaNaMgAlSiH gel. It can be observed that going farther from the GBFS particle’s boundary makes Mg drop substantially. Ca dissolves from the particles, and it enriches the outer gel. This leaves a Mg-enriched CaNaMgAlSiH gel in the GBFS particle’s boundary (A–B, C–D), similar to slag cement paste [34]. The difference of the gel’s greyscale level in the BSE image (see Figure 16) between the GBFS particle boundary (A–B, C–D) and far from that boundary is attributed to the different nanoporosities of these two regions, a higher nanoporosity of CaNaMgAlSiH gel than that of CaNaAlSiH gel was indicated.

It can also be seen that the Ca and Si show the same diffusion length scale. Mg is a bit shorter, and Al has shortest diffusion distance (lowest mobility). Na also has been found in higher amounts around rims of GBFS particles. 

**Figure 17:**
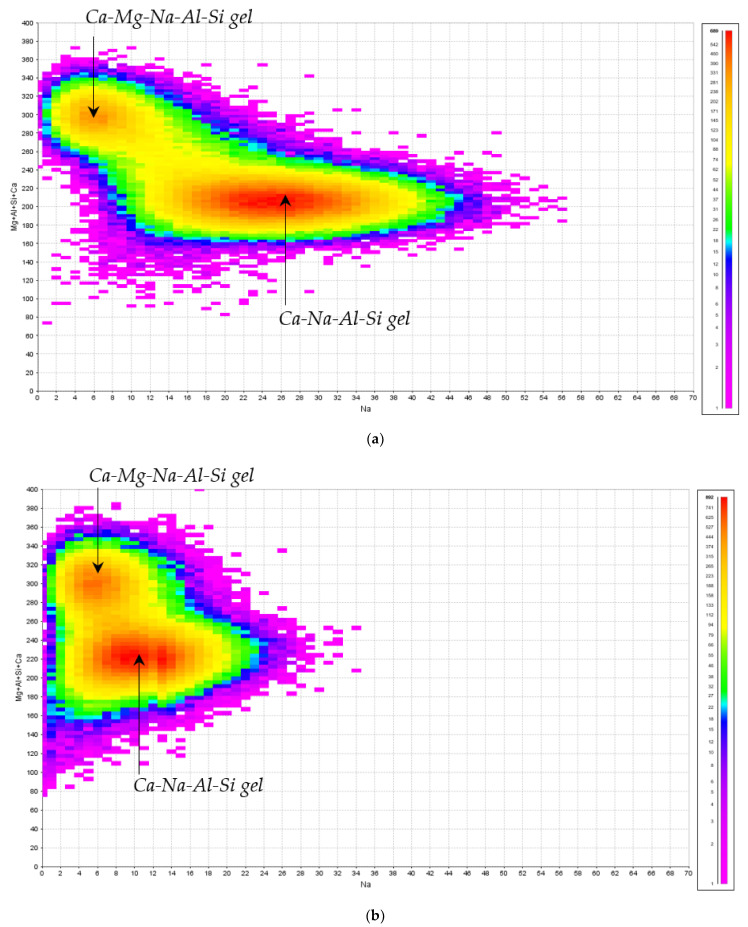
(**a**) Density plot of Na and (Mg + Al + Si + Ca) to extract groups of pixels for Ca-Na-Al-Si gel and Ca-Mg-Na-Al-Si gel in paste S100, sealed cured for 28 days; (**b**) Density plot of Na and (Mg + Al + Si + Ca) to extract groups of pixels for Ca-Na-Al-Si gel and Ca-Mg-Na-Al-Si gel in paste S100 cured and sealed for 28 days.

The Na/Si ratio is significantly dependent on the curing conditions. The authors have previously reported the significant effect of Na^+^ loss on the pore-solution composition [35]. The results presented in the current study show that the effect of Na^+^ loss is not limited to the pore-solution composition of the unsealed pastes but can also significantly affect the chemical composition of the reaction products (see 7 days and 28 days results in Table 6). This is supported by the PARC density plots of Na versus Mg + Al + Si + Ca (see Figure 17) of sealed and unsealed samples. Na is lower in the gel of unsealed cured samples compared to the sealed samples. On the other hand, the Ca/Si ratio increases from the age of 1 day to 28 days, regardless of the curing method (Table 6). This implies that the dissolution of GBFS continues in this period.

**Figure 18:**
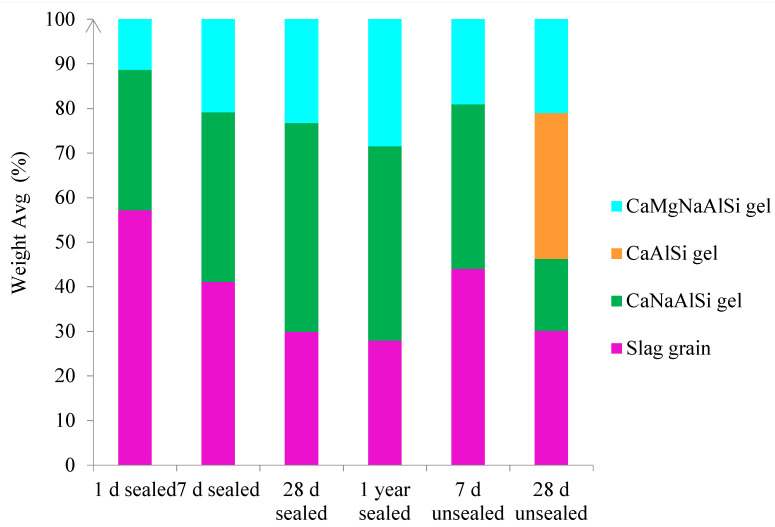
The effect of curing conditions (unsealed and sealed) on the evolution of the phases and their weight proportions in paste S100.

The evolution of the phase proportions of paste S100 is presented in Figure 18. The evolution of the phase proportions is accompanied by the compositional evolution (Table 6). Therefore, it should be noted that the gels shown in Figure 18 have a changed composition over time.

A comparison of the phase proportions of sealed cured samples at 28 days and 1 year shows that there is no further reaction of GBFS. This is supported by the strength development of this paste in previous authors’ studies [36]. It was observed that strength did not change at 1 year compared to 28 days. This suggests that the degree of reaction of GBFS is limited and reaches its maximum in the first 28 days for the studied conditions. Although the evolution of the phase proportions of paste S100 did not occur after 28 days, there still might be an element exchange, such as between Ca and Na.

In unsealed cured specimens, a CaAlSi (orange coded) phase is found at 28 days (see Figure 15), with a lower Na uptake in the gel compared to the sealed cured paste (see Table 6). This gel transformation is attributed to the loss of Na^+^ in unsealed curing conditions, which is explained in [35]. This phase, CaAlSi (orange coded), was not observed in sealed cured specimens until 1 year of curing. 

This gel transformation is attributed to the loss of Na^+^ in unsealed curing conditions, which is explained in [35]. This phase, CaAlSi (orange coded), was not observed in sealed cured specimens until 1 year of curing.

#### 3.2.2. Binary GBFS-FA Paste (S50)

**Figure 19:**
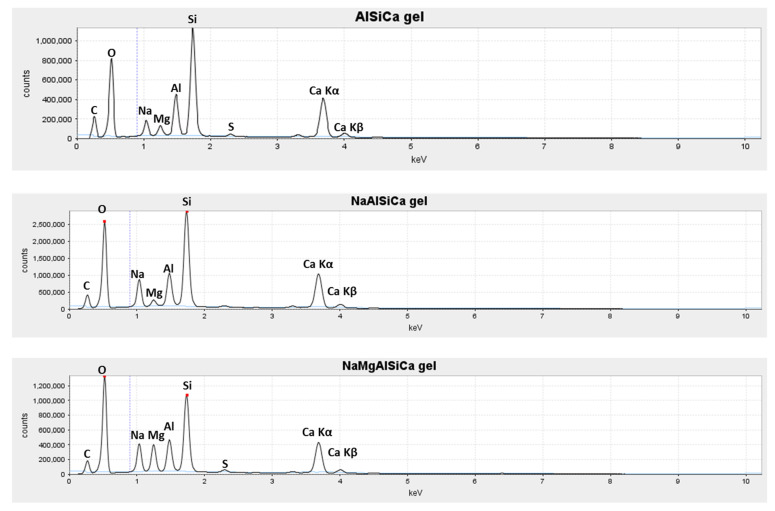
PARC sum spectrum from compound domain of CaAlSi gel, CaNaAlSi gel, and CaMgNaAlSi gel in S50.

**Figure 20:**
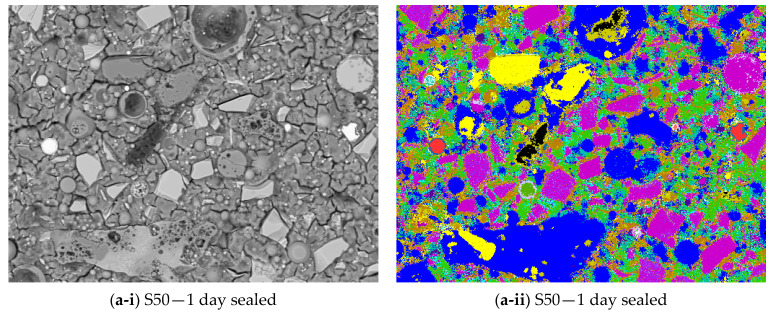

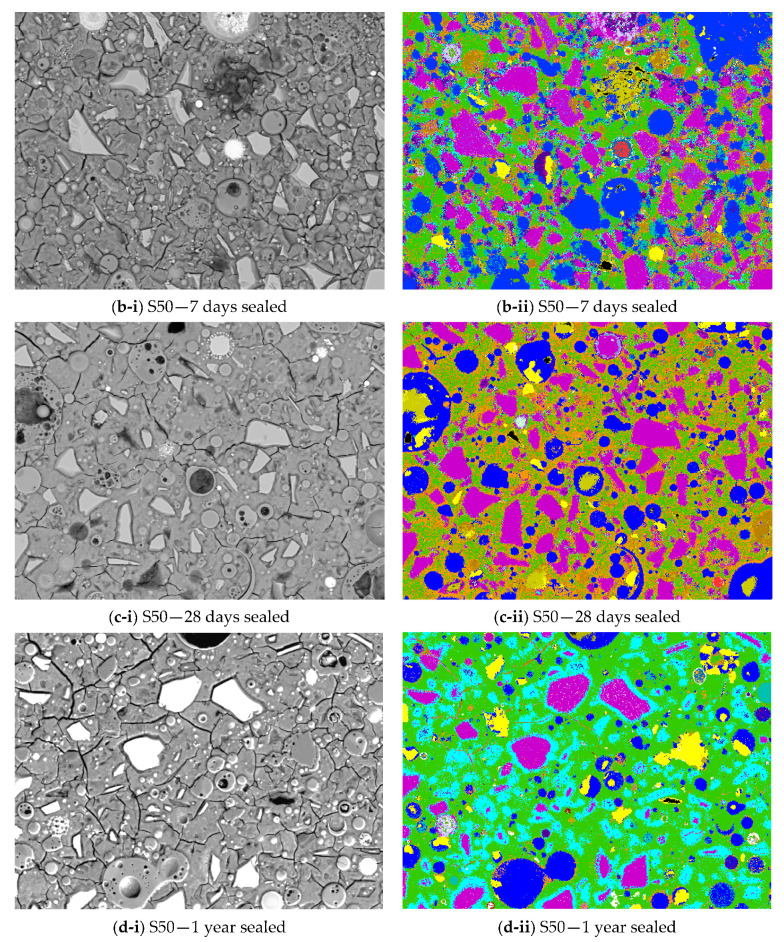

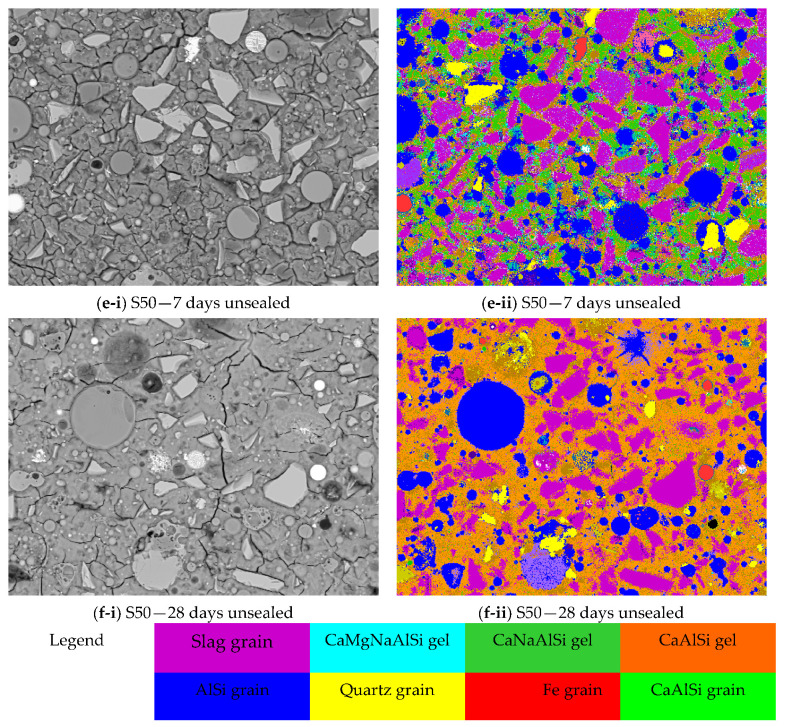
SEM-BSE images (**a–f-i**) of the microstructure of paste S50 at different curing periods, with mapped compound domains of different phases (**a–f-ii**). Image width = 250 µm.

The sum spectra of three gels (CaNaAlSi, CaMgNaAlSi and CaAlSi) observed in paste S50 sealed cured for 28 days are depicted in Figure 19. The distribution of the main reaction products is also visualized in Figure 20. The backscattered electron images of the polished sections are shown in Figure 20a–f-i, with the corresponding PARC maps presented in Figure 20a–f-ii. 

**Figure 21:**
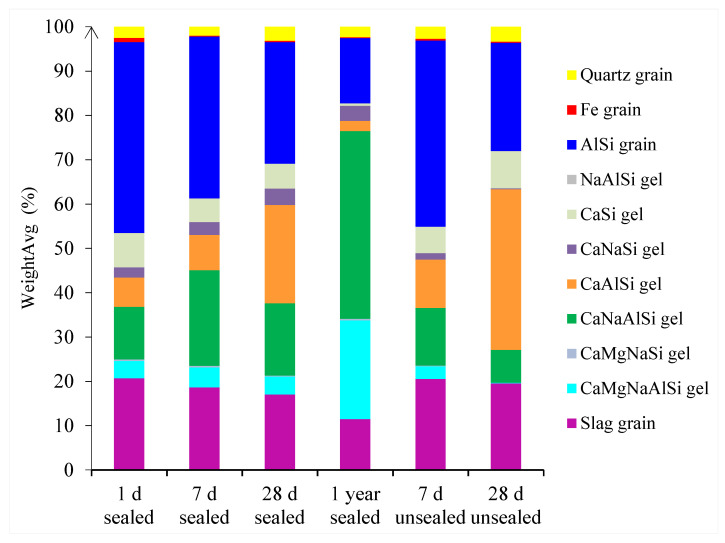
The effect of curing conditions (unsealed and sealed) on the evolution of the phases and their weight proportions in pastes S50. Note that AlSi grain includes all FA phases.

It can be observed that the reaction products appear to be very heterogeneous (Figure 20a–c,e,f-ii) at an early age and less heterogeneous at later ages of reaction (Figure 20d-ii). The composition of the reaction products in paste S50 determined by PARC is presented in Table 7. The Ca/Si ratio of the CaNaAlSi gel decreases by a factor of two in paste S50 compared to paste S100, which is proportional to its GBFS content. Besides the differences in Na, Mg, and Ca, substantial differences in the proportions of different reaction products can be seen between pastes S50 and S100 (Figure 18 vs. Figure 21). 

Figure 21 shows the effect of curing time (1 day, 7 days, 28 days, and 1 year) and curing conditions (unsealed and sealed) on the evolution of the reaction products and their proportions in pastes S50. It can be seen that a significant transformation of the reaction products has occurred during the considered times of curing.

It seems that while the GBFS dissolution has the main contribution to reactions in the early ages, FA particles (glassy AlSi grains) react at later ages. The presence of FA in the paste S50 leads to a higher Al content and consequently the formation of CaAlSi gel (Table 7). This is in contrast to paste S100, where Al is mainly incorporated in the CaMgNaAlSi gel (Table 6). Table 7 also shows, similar to paste S100, the Na content varies significantly depending on the curing conditions in the paste S50. However, the Na content in the sealed cured paste S50 (see Table 7) is constant during the entire curing regime, while for paste S100, Na decreases with elapse of curing time (see Table 6). This suggests a lower Na-binding capacity of pastes S100 with GBFS as the single precursor. 

The Na-binding capacity of FA/GBFS paste (S50) is enhanced due to the presence of FA. Namely, the silanol groups are the main adsorbing sites for alkalis. The lower the Ca/(Al + Si) is, the higher the number of silanol groups will be in the gel. This implies that more alkalis will be incorporated in C-(N-)A-S-H with a lower Ca/(Al + Si) ratio, such as in paste S50, that have a lower CaO content and a higher SiO_2_ content than paste S100.

In Figure 21, it can be also seen that CaAlSi’s gel weight increases up to 28 d and then decreases in the sealed cured specimen. What is happening here is that the primary gel composition is changing over time due to the kinetics of the dissolution of the raw materials (FA, GBFS) in certain alkaline and curing conditions. It is assumed that due to the slow rate of FA dissolution, the formation of CaAlSi gel will first take place at an early age (up to 28 d) when Na^+^ is largely present in the pore solution. At later ages, Na^+^ will be bound in the main reaction product, changing the composition of the primary reaction product from CaAlSi gel to CaNaAlSi gel.

### 3.3. Degree of Reaction

**Figure 22:**
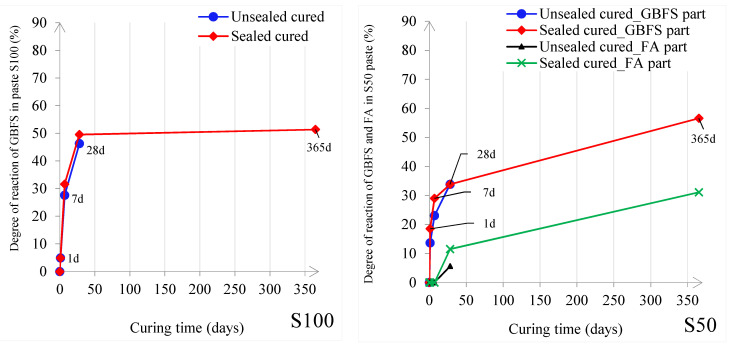
Degree of reaction of paste S100 (**left**) and paste S50 (**right**).

In both sealed and unsealed S100 pastes, the reaction rate is faster during the first 28 days (see Figure 22) compared to later ages (between 28 days and 1 year). The alkaline activation of GBFS results in the formation of a significant amount of reaction products, as can be observed from Figure 15a-i. Although the final reaction degree was nearly the same in sealed and unsealed conditions, the element ratios of reaction products are different (Table 6). It should be noted that the calculated degree of GBFS reaction is comparable with the one reported by Brough et al. [37]. 

In paste S50, GBFS has a larger reaction degree at 1 day, increasing only a little up to 28 days. However, for S50, the reaction degree of GFBS increases until the end of the tests, which is in contrast to the paste S100, in which the reaction rates significantly decreased after 28 days. In S50, the reaction of FA starts only at later ages (at 7 days), and it is negligible before that. In S50, FA has a very small reaction degree that reaches only 30% after 1 year in sealed conditions. Due to the very low reactivity of AlSi grains, the boundary layer is not formed around these grains (see Figure 20). In paste S50, FA (glassy AlSi particles) are embedded in a “homogeneous” CaNaAlSi gel. This gel is denser than the gel in the transitional zone (CaMgNaAlSi gel). This transitional zone is marked by strong compositional gradients between the GBFS and the “green” gel, which is shown in Figure 16. Since the CaMgNaAlSi gel is more porous than the CaNaAlSi gel (see Figure 16), GBFS dissolution is allowed further with time.

Also, the reaction degree of sealed and unsealed conditions is different (that is in contrast to observations made for GBFS in paste S100), probably due to different Na-binding capacities of FA and GBFS in paste S50 than of only the GBFS in paste S100. Furthermore, the low alkaline conditions and ambient curing temperature used in this study (when compared to literature [38,39]) are also possible reasons for a moderate degree of FA reaction in these samples pastes S50 at 7 days and 28 days. The degree of reaction of FA in unsealed conditions seems to be smaller than in sealed conditions. The degree of reaction of FA is mainly dependent on the reactivity of AlSi grain, NaAlSi grain, and CaAlSi grain. Quartz and Fe grain do not contribute to FA reactivity (Figure 21).

### 3.4. Bulk Chemistry

The bulk compositions from XRF analyses are shown in Table 8 and Table 9 for the S100 and S50 pastes, respectively. No specific change of weight fractions of MgO, Al_2_O_3_, SiO_2_, SO_3_, K_2_O, and CaO can be observed with the change of curing time or curing conditions in both pastes. There is also no significant difference between these weight fractions in the sealed and unsealed specimens. Meanwhile, a lower amount of Na_2_O is observed in unsealed samples. The results also show that Na_2_O content is similar in sealed cured samples at all curing ages, while it is decreasing in unsealed cured samples with time. It is important to note that the full consistency of XRF results with PARC with regard to bulk composition was not obtained (Table 8, Table 9, Table 10 and Table 11). This is most likely due to the imperfect mixing of all elements from GBFS and/or from FA. 

The lower amount of Na_2_O content in the unsealed cured samples (in both S50 and S100) at 28 days can be explained by the fact that the pore solution is not in equilibrium with the RH of the curing room atmosphere in these specimens from day 1 [35]. Due to the RH gradient between the sample and curing room atmosphere, water condensation on the samples causes dilution of the pore fluid. Consequently, the initial equilibrium between the pore fluid and the gel cannot be maintained due to dilution. This causes the gel to re-equilibrate by releasing Na^+^ into the pore fluid until the pore fluid becomes in equilibrium with the outside RH. It is interesting that the Na_2_O content and its changes are similar in both S100 and S50 pastes, which is because the same RH was imposed on samples. The rest of the oxides have different fractions in S100 and S50 that are consistent with the level of GBFS replacement. 

Finally, it can be seen that the bulk composition of the samples (S50 and S100) stays constant in both analyses, XRF (Table 8 and Table 9) and PARC (Table 10 and Table 11), while the phase proportions change with time (see Figure 18 and Figure 21).

### 3.5. Phase Mineralogy

XRD tests were only performed on S50 paste that has 50 wt% of FA and hence contains crystalline phases. These phases are quantified with the XRD-Rietveld analysis. Quantification of the reaction products with XRD was not possible for the S100 paste due to its amorphous nature. However, PARC showed to be a very useful technique in the separation of the reacted and unreacted phases and also enabled calculation of the degree of reaction of the S100 samples (Figure 22). 

**Figure 23:**
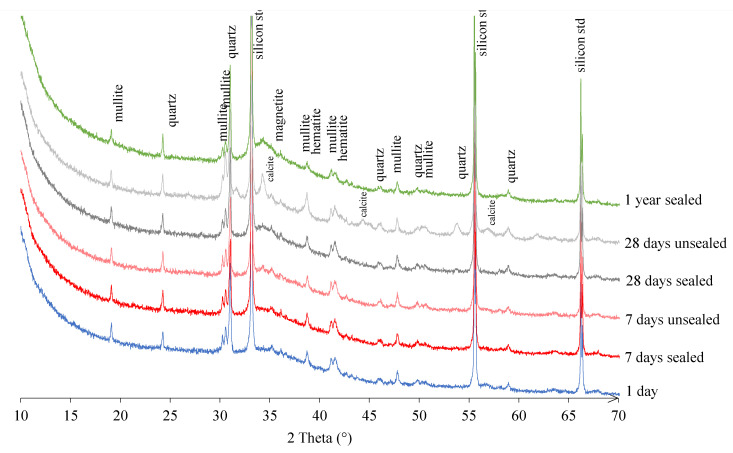
XRD diffractograms showing the phases in paste S50 for sealed and unsealed curing conditions.

**Figure 24:**
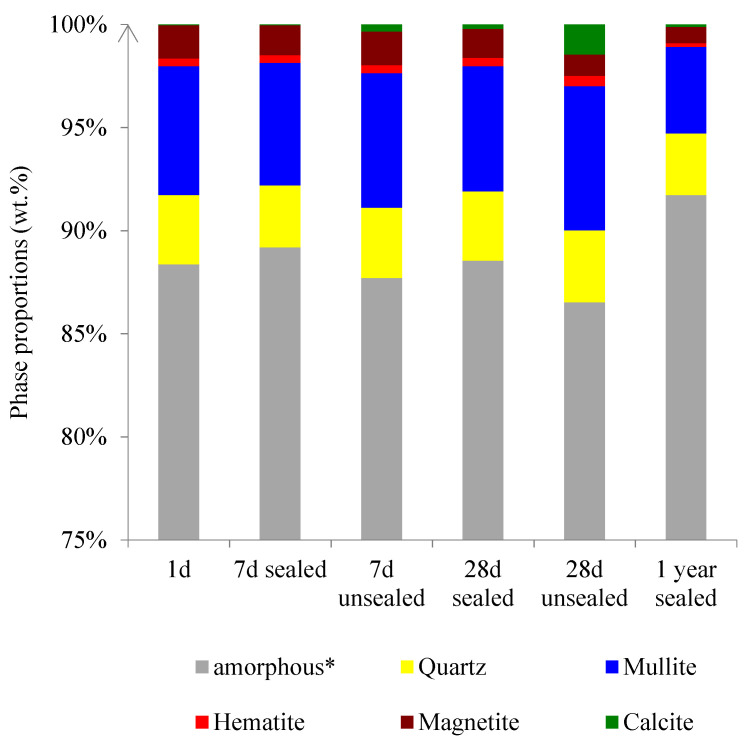
Quantitative phase analysis with the Rietveld method for paste S50 as a function of time and curing conditions. * stands for proportion of amorphous phase when excluding the internal standard in QXRD calculations.

Figure 23 shows the obtained XRD diffractograms as a function of curing time and curing conditions. The broad humps beneath the peaks of the crystalline phases of XRD diffractograms indicate the presence of an amorphous phase (Figure 23), which is quantified in Figure 24. This hump becomes more pronounced with the presence of nanocrystalline phases, such as Ca-Na-Al-Si-H (around 34 2 Theta), which is the main reaction product of the studied alkali-activated pastes. This hump is most visible in the XRD diffractogram for the sample sealed cured for 1 year. 

Figure 24 shows that curing conditions affect the mineralogical composition and the content of the phases in the samples S50. The amorphous phase content of the sealed cured pastes is similar at 1, 7, and 28 days, but it has increased after 1 year. While the amount of quartz is almost constant during 1 year of reaction (Figure 24), the mullite seems to be decreasing after 28 days. A decrease in the amorphous content can also be observed in unsealed cured samples that is due to the formation of calcite. The calcite content increases in the unsealed cured samples with the curing age. The calcite was formed due to superficial carbonation of the unsealed cured samples, as described in [35]. 

## 4. Conclusions

This study was aimed at investigating the effect of curing conditions on the microstructure evolution and chemistry (the compositions, proportions, and distribution of reaction products and unreacted material) of single and binary alkali-activated FA/GBFS pastes. This was achieved by using the PhAse Recognition and Characterization (PARC) software for the first time with these materials.

Based on the previous experience of the authors, two mixtures were chosen with the emphasis on the type of precursor, including a single system (alkali-activated GBFS, referred to as S100) and a binary system (alkali-activated GBFS + FA with a 50%:50% weight ratio, referred to as S50). The following conclusions can be drawn from the experimental observations:

- The curing conditions had a clear effect on the composition and amount of the reaction products. XRF and PARC results showed that sealing of the alkali-activated samples results in a higher Na^+^ uptake in the gels compared to the unsealed cured samples. This effect is most dominant at later ages (28 days of curing). The effect of curing conditions was more pronounced in the binary system. In addition to the chemical modifications, the mineralogy of the binary paste was also affected by the curing conditions. The formation of calcite was observed in unsealed cured samples at all investigated ages. The curing conditions are critical from a durability point of view for concrete. Sealed curing conditions will certainly contribute to durable alkali-activated FA/GBFS materials.
- The three main phases in paste S100 after 28 days of sealed curing were unreacted slag particles, CaNaAlSi gel and CaMgNaAlSi gel. In comparison, paste S50 consisted of more phases including quartz, mullite, hematite and magnetite in addition to amorphous phases (i.e., unreacted GBFS and FA, and gel phases than in paste S100). The Ca/Si ratio of the CaNaAlSi gel was lower by a factor of two in paste S50 compared to paste S100, which seems to be proportional to the GBFS content.
- Curing conditions have a significant influence on the chemical composition of the phases in the microstructure of alkali-activated pastes. This effect is the most dominant after 28 days of curing, while at 7 days, no significant change was encountered. The different chemical composition is due to the Na^+^ leaching from the samples which were unsealed cured. Instead of CNASH gel, CASH gel is formed in these samples.
- The pastes showed different reaction rates. Comparing the proportions of the phases in paste S100 at the age of 28 days and 1 year suggested that the degree of reaction of GBFS is limited and reaches its maximum at 28 days in the studied conditions. On the other hand, the phase proportions changed until 1 year in paste S50, as the reaction of the FA particles continues even after an extended period of 1 year. This indicates the role of GBFS dissolution in the early ages (28 days) and of FA in the later ages in binary alkali-activated pastes.
- In paste S100, the quantification of the reaction products was not possible with XRD due to their amorphous nature. However, PARC showed to be very useful in the identification of the reacted and the unreacted phases, which enabled calculation of the degree of reaction of the S100 samples.

## Figures and Tables

**Table 1:** Chemical compositions of FA and GBFS measured with XRF (%).

-	Na_2_O	MgO	Al_2_O_3_	SiO_2_	P_2_O_5_	S	K_2_O	CaO	TiO_2_	Fe_2_O_3_	L.O.I.
FA	0.8	1.5	23.8	56.8	0.5	0.3	1.6	4.8	1.2	7.2	1.2
GBFS	0.4	8.0	13.5	35.5	0.0	1.0	0.5	39.8	1.0	0.6	−1.3

**Table 2:** Mixture design for pastes with respect to 100 g of binder.

Mixture	FA ^a^	GBFS ^b^	m(Na_2_O)/ m(binder)	SiO_2_/ Na_2_O	Water	Activator	Curing Regime
S50	50	50	4.80	1.45	38.00	12.00	Unsealed/ Sealed
S100	0	100					

^a,b^ weight percentage with respect to raw material (FA, GBFS) content (wt%).

**Table 3:** Chemical composition of GBFS obtained with PARC (wt%).

-	Na_2_O	MgO	Al_2_O_3_	SiO_2_	P_2_O_5_	SO_3_	K_2_O	CaO	TiO_2_	MnO	Fe_2_O_3_
GBFS	0.45	8.14	13.09	35.48	0.22	2.53	0.35	38.11	1.12	0.32	0.19

**Table 4:** Chemical composition of phase domains of FA particles obtained with PARC (wt%).

-	Density (g/cm^3^)	Weight Avg	Na_2_O	MgO	Al_2_O_3_	SiO_2_	P_2_O_5_	SO_3_	K_2_O	CaO	TiO_2_	MnO	Fe_2_O_3_
Quartz (SiO_2_)	2.62	8.50	0.31	0.09	0.09	98.35	0.14	0.63	0.00	0.00	0.00	0.00	0.39
Hematite and Magnetite ^1^	5.74	0.97	0.33	0.46	0.76	3.04	0.00	0.00	0.20	0.59	0.00	0.36	94.27
AlSi_grain	2.44	76.21	0.88	0.64	31.51	58.67	0.48	0.26	1.74	0.87	1.11	0.03	6.37
NaAlSi_grain	2.44	1.91	5.36	0.78	25.58	61.24	0.57	0.16	2.03	0.60	0.58	0.2	2.90
KAlSi_grain	2.44	1.51	1.07	0.20	19.82	66.40	0.31	0.20	10.45	0.30	0.30	0.03	0.95
CaAlSi_grain	2.44	2.51	0.00	0.37	35.18	41.74	0.70	0.15	0.00	17.45	0.56	0.00	3.85
MgAlSi_grain	2.44	1.99	1.07	9.44	20.28	57.13	0.78	0.14	1.22	1.25	0.61	0.16	7.94
FeAlSi_grain	2.44	0.92	0.56	1.04	14.60	39.80	0.23	0.05	0.98	0.71	1.12	0.05	40.85
TiAlSi_grain	2.44	0.86	1.27	1.22	29.71	43.39	0.00	0.00	1.51	2.77	14.45	1.24	4.45
CaMgAlSi_grain	2.44	1.27	0.14	8.24	20.64	39.17	2.34	0.00	0.02	21.03	0.53	0.11	7.78
MgFe_grain	2.44	1.47	0.01	6.67	1.86	0.57	0.04	0.03	0.01	0.39	0.11	0.81	89.51
P_grain	2.44	0.93	0.00	6.09	11.6	17.14	24.01	0.07	0.30	36.60	0.88	0.00	3.31
Calcite (CaCO_3_)	2.71	0.29	0.00	0.00	0.82	0.43	1.54	2.33	0.00	94.38	0.00	0.00	0.49
Dolomite (CaMg(CO_3_)_2_)	2.87	0.12	0.00	32.17	1.30	2.09	0.97	3.75	0.07	57.65	0.09	0.14	1.78
Al_2_O_3_	4.00	0.54	0.08	0.00	91.84	5.37	0.45	0.02	0.07	0.31	1.02	0.02	0.84

^1^ PARC does not distinguish between Hematite and Magnetite, so they appear combined in the results.

**Table 5:** Chemical composition of FA obtained with PARC (wt%).

-	Na_2_O	MgO	Al_2_O_3_	SiO_2_	P_2_O_5_	SO_3_	K_2_O	CaO	TiO_2_	MnO	Fe_2_O_3_
FA	1.05	1.13	24.99	60.46	0.59	0.38	1.70	2.21	1.41	0.06	6.02

**Table 6:** Atomic ratios in the reaction products by PARC analysis in pastes S100.

Paste S100											
Reaction products	CaNaAlSi				CaMgNaAlSi				CaAlSi		
Atomic ratio	Na/Si	Mg/Si	Al/Si	Ca/Si	Na/Si	Mg/Si	Al/Si	Ca/Si	Na/Si	Al/Si	Ca/Si
Standard deviation	±0.003	±0.001	±0.003	±0.016	±0.020	±0.017	±0.002	±0.025	±0.007	±0.006	±0.027
1 day sealed	0.37	0.11	0.25	0.61	0.43	0.34	0.36	0.45	−	−	−
7 days sealed	0.37	0.11	0.27	0.64	0.31	0.53	0.46	0.66	−	−	−
28 days sealed	0.33	0.10	0.26	0.84	0.31	0.66	0.52	0.84	−	−	−
1 year sealed	0.29	0.10	0.24	0.85	0.25	0.69	0.51	0.83	−	−	−
7 days unsealed	0.32	0.10	0.26	0.60	0.27	0.49	0.44	0.61	−	−	−
28 days unsealed	0.12	0.11	0.25	0.69	0.08	0.41	0.40	0.73	0.06	0.18	0.71

**Table 7:** Atomic ratios in the reaction products by PARC analysis in paste S50.

Paste S50											
Reaction products	CaNaAlSi				CaMgNaAlSi				CaAlSi		
Atomic ratio	Na/Si	Mg/Si	Al/Si	Ca/Si	Na/Si	Mg/Si	Al/Si	Ca/Si	Na/Si	Al/Si	Ca/Si
Standard deviation	±0.002	±0.002	±0.002	±0.007	±0.033	±0.027	±0.011	±0.024	±0.001	±0.002	±0.002
1 day sealed	0.33	0.10	0.26	0.30	0.35	0.29	0.32	0.28	0.19	0.30	0.37
7 days sealed	0.35	0.08	0.29	0.34	0.41	0.37	0.45	0.41	0.19	0.35	0.47
28 days sealed	0.30	0.09	0.31	0.49	0.33	0.30	0.37	0.49	0.18	0.36	0.46
1 year sealed	0.40	0.05	0.30	0.50	0.47	0.37	0.38	0.51	0.11	0.55	0.35
7 days unsealed	0.30	0.08	0.28	0.33	0.41	0.29	0.43	0.31	0.16	0.29	0.38
28 days unsealed	0.25	0.09	0.30	0.48	0.26	0.28	0.35	0.47	0.14	0.31	0.49

**Table 8:** Bulk chemistry obtained with XRF for the paste S100.

S100	1 Day Sealed	7 Days Sealed	28 Days Sealed	1 Year Sealed	7 Days Unsealed	28 Days Unsealed
Na_2_O	4.48	4.80	4.45	5.12	3.96	2.76
MgO	6.00	6.11	5.89	6.71	6.29	6.02
Al_2_O_3_	10.09	10.35	9.98	10.02	10.59	10.32
SiO_2_	33.90	34.70	33.71	36.42	35.44	34.58
P_2_O_5_	0.00	0.00	0.00	0.00	0.00	0.00
S	1.44	1.44	1.43	1.60	1.32	1.20
K_2_O	0.42	0.41	0.42	0.33	0.40	0.38
CaO	40.53	39.62	41.24	37.40	39.29	41.76
TiO_2_	1.39	1.22	1.40	1.17	1.31	1.43
MnO	0.36	0.31	0.35	0.32	0.33	0.38
Fe_2_O_3_	0.69	0.66	0.68	0.61	0.66	0.67

**Table 9:** Bulk chemistry obtained with XRF for the paste S50.

S50	1 Day Sealed	7 Days Sealed	28 Days Sealed	1 Year Sealed	7 Days Unsealed	28 Days Unsealed
Na_2_O	5.09	5.12	4.46	4.56	4.78	3.88
MgO	3.87	4.07	3.78	4.16	4.21	4.50
Al_2_O_3_	15.42	15.08	13.71	14.41	15.36	15.49
SiO_2_	43.27	43.38	41.00	45.74	44.12	44.83
P_2_O_5_	0.31	0.32	0.30	0.23	0.32	0.34
S	1.07	1.10	1.17	1.12	1.00	0.96
K_2_O	0.85	0.86	0.93	0.81	0.82	0.80
CaO	22.50	22.92	25.89	22.58	22.39	22.47
TiO_2_	1.34	1.36	1.59	1.30	1.26	1.34
MnO	0.20	0.21	0.26	0.21	0.20	0.21
Fe_2_O_3_	4.94	4.82	6.03	4.73	4.65	4.58

**Table 10:** Bulk chemistry obtained with PARC for the paste S100.

S100	1 Day Sealed	7 Days Sealed	28 Days Sealed	1 Year Sealed	7 Days Unsealed	28 Days Unsealed
Na_2_O	4.71	4.92	4.90	4.35	4.32	1.77
MgO	7.03	7.10	6.82	7.17	7.15	6.68
Al_2_O_3_	12.09	12.06	11.51	10.84	12.26	11.60
SiO_2_	30.04	31.03	33.62	33.87	31.06	33.49
P_2_O_5_	0.32	0.30	0.24	0.24	0.31	0.31
SO_3_	1.68	1.95	1.72	1.66	1.60	1.24
K_2_O	0.54	0.47	0.36	0.31	0.48	0.34
CaO	41.09	40.44	39.22	39.80	41.23	42.95
TiO_2_	1.01	0.99	1.02	1.01	1.03	1.03
MnO	0.29	0.29	0.29	0.29	0.30	0.28
Fe_2_O_3_	0.40	0.44	0.31	0.48	0.25	0.33

**Table 11:** Bulk chemistry obtained with PARC for the paste S50.

S50	1 Day Sealed	7 Days Sealed	28 Days Sealed	1 Year Sealed	7 Days Unsealed	28 Days Unsealed
Na_2_O	4.26	4.54	4.13	6.49	3.61	2.55
MgO	4.83	5.13	4.51	4.42	5.26	4.84
Al_2_O_3_	16.46	17.41	17.06	15.21	16.87	16.30
SiO_2_	42.88	42.55	40.30	44.23	43.22	43.21
P_2_O_5_	0.61	0.61	0.55	0.55	0.61	0.55
SO_3_	1.22	1.40	1.85	1.71	1.46	1.62
K_2_O	1.09	1.05	1.00	0.98	1.03	0.84
CaO	23.77	23.44	26.83	22.69	23.80	26.73
TiO_2_	0.94	1.03	0.91	0.98	1.05	0.91
MnO	0.21	0.19	0.17	0.23	0.21	0.20
Fe_2_O_3_	3.71	2.39	2.44	2.61	2.90	2.24

## Data Availability

Data sharing is not applicable to this article.
